# Current Awareness on Comparative and Functional Genomics

**DOI:** 10.1002/cfg.117

**Published:** 2002-04


**Comparative and Functional Genomics**
*Comp. Funct. Genom.* 2002; **3:** 211–218
**DOI**:10.1002/cfg.117

### Current awareness on comparative and functional genomics

CURRENT AWARENESS - USE THE LINKS ONLINE

### 1 Reviews & symposia

Adam BL, Vlahou A, Semmes OJ, Wright GL*. 2001. *Eastern Virginia Med Sch, Dept Microbiol & Mol Cell Biol, 700 West Olney Rd, Norfolk, Va 23501, USA. Proteomic approaches to biomarker discovery in prostate and bladder cancers (Review). *Proteomics* **1:** (10) 1264.

Aitman TJ. 2001. Hammersmith Hosp, MRC Ctr Clin Sci, Physiol Genomics & Med Grp, London W12 0NN, England. Science, medicine, and the future: DNA microarrays in medical practice (Review). *Br Med J* **323:** (7313) 611.

Alizadeh AA, Ross DT, Perou CM, Van de Rijn M*. 2001. *Stanford Univ, Med Ctr, Dept Pathol, 300 Pasteur Dr, Stanford, Ca 94305, USA. Towards a novel classification of human malignancies based on gene expression patterns. *J Pathol* **195:** (1) 41.

Barrett AJ, Rawlings ND, O'Brien EA. 2001. Babraham Inst, Mol Enzymol Lab, Cambridge CB2 4AT, England. The MEROPS database as a protease information system. *J Struct Biol* **134:** (2-3) 95.

Bubendorf L, Nocito A, Moch H, Sauter G*. 2001. *Univ Basel Hosp, Inst Pathol, Schonbeinstr 40, CH-4003 Basel, Switzerland. Tissue microarray (TMA) technology: Miniaturized pathology archives for high-throughput *in situ* studies. *J Pathol* **195:** (1) 72.

Copeland NG, Jenkins NA, Court DL. 2001. NCI, Ctr Canc Res, Mouse Canc Genet Program, Frederick, Md 21702, USA. Recombineering: A powerful new tool for mouse functional genomics (Review). *Nat Rev Genet* **2:** (10) 769.

Craven RA, Banks RE*. 2001. *St James's Univ Hosp, Imperial Canc Res Fund, Canc Med Res Unit, Beckett St, Leeds LS9 7TF, England. Laser capture microdissection and proteomics: Possibilities and limitation (Review). *Proteomics* **1:** (10) 1200.

Dhiman N, Bonilla R, O'Kane D, Poland GA*. 2001. *Mayo Clin & Mayo Fdn, Mayo Vaccine Res Grp, Dept Internal Med, Clin Pharmacol Unit, Rochester, Mn 55905, USA. Gene expression microarrays: A XXIst century tool for directed vaccine design (Review). *Vaccine* **20:** (1-2) 22.

Dobrindt U, Hacker J*. 2001. *Univ Wurzburg, Inst Mol Infekt Biol, Rontgenring 11, DE-97070 Wurzburg, Germany. Whole genome plasticity in pathogenic bacteria. *Curr Opin Microbiol* **4:** (5) 550.

Dua K, Williams TM, Beretta L*. 2001. *Univ Michigan, Sch Med, Dept Microbiol & Immunol, 1150 West Med Ctr Dr, Ann Arbor, Mi 48109, USA. Translational control of the proteome: Relevance to cancer (Review). *Proteomics* **1:** (10) 1191.

Evans WE, Johnson JA. 2001. St Jude Children's Hosp, Dept Pharmaceut Sci, 332 Nth Lauderdale St, Memphis, Tn 38105, USA. Pharmacogenomics: The inherited basis for interindividual differences in drug response. *Annu Rev Genomic Hum Genet* **2:** 9.

Fiehn O. 2001. Max-Planck-Inst Molec Plant Physiol, DE-14424 Potsdam, Germany. Combining genomics, metabolome analysis, and biochemical modelling to understand metabolic networks (Review). *Comp Funct Genom* **2:** (3) 155.

Flajnik MF, Kasahara M. 2001. Grad Univ Adv Studies, Sch Adv Sci, Dept Biosyst Sci, Hayama 240 0193, Japan. Comparative genomics of the MHC: Glimpses into the evolution of the adaptive immune system (Review). *Immunity* **15:** (3) 351.

Frantz GD, Pham TQ, Peale FV, Hillan KJ. 2001. Genentech Inc, Dept Pathol, MS 6, 1 DNA Way, Sth San Francisco, Ca 94080, USA. Detection of novel gene expression in paraffin-embedded tissues by isotopic *in situ* hybridization in tissue microarrays. *J Pathol* **195:** (1) 87.

Glusman G, Rowen L, Lee I, Boysen C, Roach JC, Smit AFA, Wang K, Koop BF, Hood L*. 2001. *Inst Syst Biol, 4225 Roosevelt Way NE, Seattle, Wa 98105, USA. Comparative genomics of the human and mouse T-cell receptor loci (Review). *Immunity* **15:** (3) 337.

Glynne RJ, Watson SR. 2001. Eos Biotechnol, 225A Gateway Blvd, Sth San Francisco, Ca 94080, USA. The immune system and gene expression microarrays: New answers to old questions. *J Pathol* **195:** (1) 20.

Greenberg SA. 2001. Brigham & Women's Hosp, Dept Neurol, Neuromuscular Div, 75 Francis St, Boston, Ma 02115, USA. DNA microarray gene expression analysis technology and its application to neurological disorders. *Neurology* **57:** (5) 755.

Hess KR, Zhang W, Baggerly KA, Stivers DN, Coombes KR. 2001. Univ Texas, MD Anderson Canc Ctr, Dept Biostat, 1515 Holcombe Blvd, Box 447, Houston, Tx 77030, USA. Microarrays: Handling the deluge of data and extracting reliable information (Review). *Trends Biotechnol* **19:** (11) 463.

Hondermarck H, Vercoutter-Edouard AS, Revillion F, Lemoine J, El Yazidi-Belkoura I, Nurcombe V, Peyrat JP. 2001. Univ Lille 1, UPRES EA 1033, Dev Biol Lab, Bat SN3, FR-59650 Villeneuve d'Ascq, France. Proteomics of breast cancer for marker discovery and signal pathway profiling (Review). *Proteomics* **1:** (10) 1216.

Hutter G, Sinha P*. 2001. *Univ Klinikum Berlin, Charite, Inst Lab Med & Pathobiochem, Campus Charite Mitte, Schumannstr 20-21, DE-10117 Berlin, Germany. Proteomics for studying cancer cells and the development of chemoresistance (Review). *Proteomics* **1:** (10) 1233.

Issaq HJ. 2001. SAIC-Frederick, NIC-Frederick, POB B, Frederick, Md 21702, USA. The role of separation science in proteomics research (Review). *Electrophoresis* **22:** (17) 3629.

Kajava AV. 2001. NIH/CIT, Ctr Mol Modeling, Bldg 12A, Room 2011, Bethesda, Md 20892, USA. Proteins with repeated sequence: Structural prediction and modeling (Review). *J Struct Biol* **134:** (2-3) 132.

Kelly DE, Lamb DC, Kelly SL. 2001. Univ Wales, Inst Biol Sci, Abersystwyth SY23 3DA, Wales. Genome-wide generation of yeast gene deletion strains (Review). *Comp Funct Genom* **2:** (4) 236.

Kettman JR, Frey JR, Lefkovits I*. 2001. *Basel Inst Immunol, Grenzacherstr 487, CH-4005 Basel, Switzerland. Proteome, transcriptome and genome: Top down or bottom up analysis? (Re-

In order to keep subscribers up-to-date with the latest developments in their field, this current awareness service is provided by John Wiley & Sons and contains newly-published material on comparative and functional genomics. Each bibliography is divided into 16 sections. 1 Reviews & symposia; 2 General; 3 Large-scale sequencing and mapping; 4 Evolutionary genomics; 5 Comparative genomics; 6 Pathways, gene families and regulons; 7 Pharmacogenomics; 8 EST, cDNA and other clone resources; 9 Functional genomics; 10 Transcriptomics; 11 Proteomics; 12 Protein structural genomics; 13 Metabolomics; 14 Genomic approaches to development; 15 Technological advances; 16 Bioinformatics. Within each section, articles are listed in alphabetical order with respect to author. If, in the preceding period, no publications are located relevant to any one of these headings, that section will be omitted.

Copyright © 2002 John Wiley & Sons, Ltd.

view). *Biomol Eng* **18:** (5) 207.
Kohler H, Murali R, Kieber-Emmons T. 2001. Univ Kentucky, Lexington, Ky 40536, USA. The hidden code in genomics: A tool for gene discovery (Review). *J Mol Recognit* **14:** (5) 269.
Korenberg MJ, David R, Hunter IW, Solomon JE. 2001. Queen's Univ, Dept Elect & Comp Engn, Kingston, Ontario, Canada K7L 3N6. Parallel cascade identification and its application to protein family prediction (Review). *J Biotechnol* **91:** (1) 35.
Le Naour F. 2001. Hop Paul Brousse, INSERM U268, FR-94807 Villejuif, France. Contribution of proteomics to tumor immunology (Review). *Proteomics* **1:** (10) 1295.
Lee RT. 2001. Partners Res Facil, Rm 279, 65 Lansdowne St, Cambridge, Ma 02139, USA. Functional genomics and cardiovascular drug discovery. *Circulation* **104:** (12) 1441.
Lewis F, Maughan NJ, Smith V, Hillan K, Quirke P. 2001. Leeds Teaching Hosp, Leeds, England. Unlocking the archive: Gene expression in paraffin-embedded tissue. *J Pathol* **195:** (1) 66.
Lievens S, Goormachtig S, Holsters M*. 2001. *State Univ Ghent, Vlaams Interuniv Inst Biotechnol, Dept Plantengenet, Vakgrp Mol Genet, BE-9000 Ghent, Belgium. A critical evaluation of differential display as a tool to identify genes involved in legume nodulation: Looking back and looking forward. *Nucleic Acids Res* **29:** (17) 3459.
Liotta LA, Kohn EC, Petricoin EF. 2001. 10 Ctr Dr, MSC 1500, Bethesda, Md 20892, USA. Clinical proteomics: Personalized molecular medicine. *JAMA* **286:** (18) 2211.
Lobenhofer EK, Bushel PR, Afshari CA*, Hamadeh HK. 2001. *NIEHS, Mol Carcinogenesis Lab, POB 12233, Res Triangle Park, NC 27709, USA. Progress in the application of DNA microarrays (Review). *Environ Health Perspect* **109:** (9) 881.
Martin DB, Nelson PS. 2001. Fred Hutchinson Canc Res Ctr, Div Human Biol, Seattle, Wa 98109, USA. From genomics to proteomics: Techniques and applications in cancer research. *Trends Cell Biol* **11:** (11) S60.
McLachlin DT, Chait BT. 2001. Rockefeller Univ, 1230 York Ave, New York, NY 10021, USA. Analysis of phosphorylated proteins and peptides by mass spectrometry (Review). *Curr Opin Chem Biol* **5:** (5) 591.
Meri S, Bauman M. 2001. Univ Helsinki, Biomedicum, Inst Biomed, Prot Chem Unit, POB 63, Haartmaninkatu 8, FI-00014 Helsinki, Finland. Proteomics: Post-translational modifications, immune responses and current analytical tools (Review). *Biomol Eng* **18:** (5) 213.
Mira A, Ochman H, Moran NA. 2001. Univ Arizona, Dept Ecol & Evolutionary Biol, Tucson, Az 85721, USA. Deletional bias and the evolution of bacterial genomes (Review). *Trends Genet* **17:** (10) 589.
Nakai K. 2001. Univ Tokyo, Inst Med Sci, Ctr Human Genome, Minato ku, 4-6-1 Shirokanedai, Tokyo 108 8639, Japan. Prediction of *in vivo* fates of protein in the era of genomics and proteomics (Review). *J Struct Biol* **134:** (2-3) 103.
Noordewier MO, Warren PV. 2001. GlaxoSmithKline Pharmaceut, Dept Bioinformatics, 1250 Sth Collegeville Rd, POB 5089, Collegeville, Pa 19426, USA. Gene expression microarrays and the integration of biological knowledge (Review). *Trends Biotechnol* **19:** (10) 412.
Nyman TA. 2001. Turku Ctr Biotechnol, Biocity, POB 123, FI-20521 Turku, Finland. The role of mass spectrometry in proteome studies (Review). *Biomol Eng* **18:** (5) 221.
Ong SE, Pandey A*. 2001. *Whitehead Inst Biomed Res, 9 Cambridge Ctr, Cambridge, Ma 02142, USA. An evaluation of the use of two-dimensional gel electrophoresis in proteomics (Review). *Biomol Eng* **18:** (5) 195.
Orengo CA, Sillitoe I, Reeves G, Pearl FMG. 2001. UCL, Dept Biochem & Mol Biol, Gower St, London WC1E 6BT, England. What can structural classifications reveal about protein evolution? (Review). *J Struct Biol* **134:** (2-3) 145.
Peale FV, Gerritsen ME. 2001. Genentech Inc, Dept Pathol, 1 DNA Way, Sth San Francisco, Ca 94080, USA. Gene profiling techniques and their application in angiogenesis and vascular development. *J Pathol* **195:** (1) 7.
Pokala N, Handel TM. 2001. Univ Calif, Dept Mol & Cell Biol, 229 Stanley Hall, Berkeley, Ca 94720, USA. Protein design: Where we were, where we are, where we're going (Review). *J Struct Biol* **134:** (2-3) 269.
Potuschak T, Doerner P. 2001. Univ Edinburgh, Inst Cell & Mol Biol, Edinburgh EH9 3JR, Scotland. Cell cycle controls: Genome-wide analysis in *Arabidopsis*. *Curr Opin Plant Biol* **4:** (6) 501.
Read TD, Gill SR, Tettelin H, Dougherty BA. 2001. Inst Genome Res, Microbial Genomics Grp, 9712 Med Ctr Dr, Rockville, Md 20851, USA. Finding drug targets in microbial genomes (Review). *Drug Discov Today* **6:** (17) 887.
Rost B. 2001. Columbia Univ, Dept Biochem & Mol Biophys, CUBIC, 630 West 168th St, New York, NY 10032, USA. Protein secondary structure prediction continues to rise (Review). *J Struct Biol* **134:** (2-3) 204.
Rowe-Magnus DA, Mazel D*. 2001. *Inst Pasteur, Dept Biotechnol, CNRS, URA 1444, Unite Programmat Mol & Toxicol Genet, 25 rue Dr Roux, FR-75724 Paris, France. Integrons: Natural tools for bacterial genome evolution. *Curr Opin Microbiol* **4:** (5) 565.
Rubin MA. 2001. Univ Michigan, Dept Pathol, 1500 East Med Ctr Dr, Room 2G332, Box 0054, Ann Arbor, Mi 48109, USA. Use of laser capture microdissection, cDNA microarrays, and tissue microarrays in advancing our understanding of prostate cancer. *J Pathol* **195:** (1) 80.
Sarto C, Deon C, Doro G, Hochstrasser DF, Mocarelli P, Sanchez JC. 2001. Univ Milan, Desio Hosp, Dept Lab Med, via Mazzini 1, IT-20033 Milan, Italy. Contribution of proteomics to the molecular analysis of renal cell carcinoma with an emphasis on manganese superoxide dismutase (Review). *Proteomics* **1:** (10) 1288.
Schmidt-Weber CB, Wohlfahrt JG, Blaser K. 2001. Swiss Inst Allergy & Asthma Res, Obere Str 22, CH-7270 Davos, Switzerland. DNA arrays in allergy and immunology (Review). *Int Arch Allergy Immunol* **126:** (1) 1.
Seow TK, Liang RCMY, Leow CK, Chung MCM*. 2001. *Natl Univ Singapore, Fac Med, Dept Biochem, SG-119260 Singapore, Rep Singapore. Hepatocellular carcinoma: From bedside to proteomics (Review). *Proteomics* **1:** (10) 1249.
Simpson AJG, De Souza SJ, Camargo AA, Brentani RR. 2001. Ludwig Inst Cancer Res, Rua Prof Antonio Prudente 109, 4th Floor, BR-10509-010 Sao Paulo, SP, Brazil. Definition of the gene content of the human genome: The need for deep experimental verification (Review). *Comp Funct Genom* **2:** (3) 169.
Srinivas PR, Srivastava S*, Hanash S, Wright GL. 2001. *NCY Div Canc Prevent, Canc Biomarkers Res Grp, 6130 Executive Blvd, Rm EPN 330F, Rockville, Md 20852, USA. Proteomics in early detection of cancer. *Clin Chem* **47:** (10) 1901.
Strausberg RL. 2001. NCI, Bethesda, Md 20892, USA. The Cancer Genome Anatomy Project: New resources for reading the molecular signatures of cancer. *J Pathol* **195:** (1) 31.
Tang CM, Moxon ER. 2001. Univ Oxford, John Radcliffe Hosp, Dept Paediat, Oxford OX3 9DU, England. The impact of microbial genomics on antimicrobial drug development. *Annu Rev Genomic Hum Genet* **2:** 259.
Tschulik A, Zatloukal K*. 2001. *Graz Univ, Inst Pathol, Auenbruggerpl 25, AU-8036 Graz, Austria. The increasing importance of tissue banks for todays genomic and proteomic research (Review) (German). *Pathologe* **22:** (5) 310.
Vihinen M. 2001. Univ Tampere, Inst Med Technol, FI-33014 Tampere, Finland. Bioinformatics in proteomics (Review). *Biomol Eng* **18:** (5) 241.
Vladutiu GD. 2001. SUNY Buffalo, Sch Med & Biomed Sci, Dept Pediat, Div Genet, 936 Delaware Ave, Buffalo, NY 14209, USA. Heterozygosity: An expanding role in proteomics (Minireview). *Mol Genet Metab* **74:** (1-2) 51.
Wallace BA, Janes RW. 2001. Univ London, Birbeck Coll, Sch Crystallog, London WC1E 7HX, England. Synchrotron radiation circular dichroism spectroscopy of proteins: Secondary structure, fold recognition and structural genomics (Review). *Curr Opin Chem Biol* **5:** (5) 567.
Ward SJ. 2001. Millenium Pharmaceut Inc, Cambridge, Ma, USA. Impact of genomics in drug discovery. *Biotechniques* **31:** (3) 626.
Wu TD. 2001. Genentech Inc, Dept Bioinformat, 1 DNA Way, MS 93, Sth San Francisco, Ca 94080, USA. Analysing gene expression data from DNA microarrays to identify candidate genes. *J Pathol* **195:** (1) 53.
Wulfkuhle JD, McLean KC, Paweletz CP, Sgroi DC, Trock BJ, Steeg PS, Petricoin EF*. 2001. *CBER/FDA, Div Therapeut Prod, Bldg 29 A, Room 2802, 8800 Rockville Pike, Bethesda, Md 20892, USA. New approaches to proteomic analysis of breast cancer (Review). *Proteomics* **1:** (10) 1205.
Zagursky RJ, Russell D. 2001. Wyeth Lederle Vaccines, 211 Bailey Rd, West Henrietta, NY 14586, USA. Bioinformatics: Use in bacterial vaccine discovery. *Biotechniques* **31:** (3) 636.
Zak NB, Shifman S, Shalom A, Darvasi A. 2001. IDgene Pharmaceut,

Copyright © 2002 John Wiley & Sons, Ltd. 

POB 34478, IL-91344 Jerusalem, Israel. Population-based gene discovery in the post-genomic era (Review). *Drug Discov Today* **6:** (21) 1111.

Zapico MEF, Ahmad US, Urrutia R*. 2001. *St Mary's Hosp, Gastroenterol Res Unit, Dept Biochem & Mol Biol, 2-445 Alfred Bldg, Rochester, Mn 55905, USA. DNA microarrays: Revolutionary insight into the living genome (Review). *Surgery* **130:** (3) 403.

### 3 Large-scale sequencing and mapping

Cai WW, Chen R, Gibbs RA*, Bradley A. 2001. *Baylor Coll Med, Dept Mol & Human Genet, Houston, Tx 77030, USA. A clone-array pooled shotgun strategy for sequencing large genomes. *Genome Res* **11:** (10) 1619.

Kawarabayasi Y, Hino Y, Horikawa H, Jin-no K, Takahashi M, Sekine M, Baba S, Ankai A, Kosugi H, Hosoyama A *et al*. 2001. Natl Inst Technol & Evaluat, 2-49-10 Nishihara, Tokyo 151 0066, Japan. Complete genome sequence of an aerobic thermoacidophilic crenarchaeon, *Sulfolobus tokodaii* strain7. *DNA Res* **8:** (4) 123.

Kerjean A, Viellefond A, Thiounn N, Sibony M, Jeanpierre M, Jouannet P. 2001. Inst Cochin Genet Mol, INSERM U257, 24 rue Faubourg St Jacques, FR-75014 Paris, France. Bisulfite genomic sequencing of microdissected cells. *Nucleic Acids Res* **29:** (21) U33.

Omura S, Ikeda H, Ishikawa J, Hanamoto A, Takahashi C, Shinose M, Takahashi Y, Horikawa H, Nakazawa H, Osonoe T, Kikuchi H, Shiba T, Sasaki Y, Hattori M. 2001. Kitasato Inst, Minato ku, 9-1 Shirokane 5 chome, Tokyo 108 8642, Japan. Genome sequencing of an industrial microorganism *Streptomyces avermitilis*: Deducing the ability of producing secondary metabolites. *Proc Natl Acad Sci U S A* **98:** (21) 12215.

Parkhill J, Wren BW, Thomson NR, Titball RW, Holden MT, Prentice MB, Sebaihia M, James KD, Churcher C, Mungall KL *et al*. 2001. Sanger Ctr, Wellcome Trust Genome Campus, Cambridge CB10 1SA, England. Genome sequence of *Yersinia pestis*, the causative agent of plague. *Nature* **413:** (6855) 523.

### 4 Evolutionary genomics

Baumbusch LO, Thorstensen T, Krauss V, Fischer A, Naumann K, Assalkhou R, Schulz I, Reuter G, Aalen RB*. 2001. *Univ Oslo, Dept Biol, Div Mol Biol, POB 1031, Blindern, NO-0315 Oslo, Norway. The *Arabidopsis thaliana* genome contains at least 29 active genes encoding SET domain proteins that can be assigned to four evolutionarily conserved classes. *Nucleic Acids Res* **29:** (21) 4319.

Liberles DA. 2001. Stockholm Univ, Dept Biochem & Biophys, Stockholm, Sweden. Evaluation of methods for determination of a reconstructed history of gene sequence evolution. *Mol Biol Evol* **18:** (11) 2040.

Miya M, Kawaguchi A, Nishida M. 2001. Nat Hist Museum & Inst, Dept Zool, Chuo ku, 955-2 Aoba cho, Chiba 260 8682, Japan. Mitogenomic exploration of higher teleostean phylogenies: A case study for moderate-scale evolutionary genomics with 38 newly determined complete mitochondrial DNA sequences. *Mol Biol Evol* **18:** (11) 1993.

Schmid KJ, Aquadro CF. 2001. Max-Planck-Inst Chem Ecol, Dept Genet & Evolut, Carl Zeiss Promenade 10, DE-07745 Jena, Germany. The evolutionary analysis of "orphans" from the *Drosophila* genome identifies rapidly diverging and incorrectly annotated genes. *Genetics* **159:** (2) 589.

### 5 Comparative genomics

Desiere F, McShan WM, Van Sinderen D, Ferretti JJ, Brussow H*. 2001. *Nestec Ltd, Nestle Res Ctr, Vers Chez-Les-Blanc, CH-1000 Lausanne 26, Switzerland. Comparative genomics reveals close genetic relationships between phages from dairy bacteria and pathogenic streptococci: Evolutionary implications for prophage-host interactions. *Virology* **288:** (2) 325.

Dorrell N, Mangan JA, Laing KG, Hinds J, Lindon D, Al Ghuesein H, Barrell BG, Parkhill J, Stoker NG, Karlyshev AV, Butcher PD, Wren BW. 2001. *Univ London, London Sch Hyg & Trop Med, Dept Infect & Trop Dis, Keppel St, London WC1E 7HT, England. Whole genome comparison of *Campylobacter jejuni* human isolates using a low-cost microarray reveals extensive genetic diversity. *Genome Res* **11:** (10) 1706.

Glaser P, Frangeul L, Buchrieser C, Rusniok C, Amend A, Baquero F, Berche P, Bloecker H, Brandt P, Chakraborty T *et al*. 2001. c/o Cossart P, Inst Pasteur, Unite Interact Bacteries Cellules, 25-28 rue Dr Roux, FR-75724 Paris, France. Comparative genomics of Listeria species. *Science* **294:** (5543) 849.

Horimoto K, Fukuchi S, Mori K. 2001. Saga Med Sch, Math Lab, 5-1-1 Nabeshima, Saga 849 8501, Japan. Comprehensive comparison between locations of orthologous genes on archaeal and bacterial genomes. *Bioinformatics* **17:** (9) 791.

Ryder CD, Smith LB, Teakle GR, King GJ*. 2001. *Hort Res Int, Dept Plant Genet & Biotechnol, Warwick CV35 9EF, England. Contrasting genome organisation: Two regions of the *Brassica oleracea* genome compared with collinear regions of the *Arabidopsis thaliana* genome. *Genome* **44:** (5) 808.

### 7 Pharmacogenomics

Alevizos I, Mahadevappa M, Zhang X, Ohyama H, Kohno Y, Posner M, Gallagher GT, Varvares M, Cohen D, Kim D, Kent R, Donoff RB, Todd R, Yung CM, Warrington JA, Wong DT*. 2001. *Harvard Univ, Sch Dent Med, Dept Oral Med & Diagnost Sci, Div Oral Biol, 188 Longwood Ave, Boston, Ma 02115, USA. Oral cancer *in vivo* gene expression profiling assisted by laser capture microdissection and microarray analysis. *Oncogene* **20:** (43) 6196.

Foxwell BMJ, Yoshimura S, Bondeson J, Brennan FM, Feldmann M. 2001. Univ London, Imperial Coll Sci Technol & Med, Sch Med, Kennedy Inst Rheumatol Div, 1 Aspenlea Rd, London W6 8LH, England. High efficiency gene transfer is an efficient way of defining therapeutic targets: A functional genomics approach. *Ann Rheum Dis* **60:** (Suppl 3) III13.

Hoffmann KF, McCarty TC, Segal DH, Chiaramonte M, Hesse M, Davis EM, Cheever AW, Meltzer PS, Morse HC, Wynn TA*. 2001. *NIH/NIAID, Immunobiol Sect, Parasit Dis Lab, 9000 Rockville Pike, Bethesda, Md 20892, USA. Disease fingerprinting with cDNA microarrays reveals distinct gene expression profiles in lethal type-1 and type-2 cytokine-mediated inflammatory reactions. *FASEB J* **15:** (11) U233.

Kan T, Shimada Y*, Sato F, Maeda M, Kawabe A, Kaganoi J, Itami A, Yamasaki S, Imamura M. 2001. *Kyoto Univ, Grad Sch Med, Dept Surg & Surg Basic Sci, Sakyo ku, Kawaracho 54, Kyoto 606 8507, Japan. Gene expression profiling in human esophageal cancers using cDNA microarray. *Biochem Biophys Res Commun* **286:** (4) 792.

Knezevic V, Leethanakul C, Bichsel VE, Worth JM, Prabhu VV, Gutkind JS, Liotta LA, Munson PJ, Petricoin EF, Krizman DB*. 2001. *NIH/NCI, Adv Technol Ctr, Pathol Lab, 8717 Grovement Circle, Gaithersburg, Md 20877, USA. Proteomic profiling of the cancer microenvironment by antibody arrays. *Proteomics* **1:** (10) 1271.

Oh JMC, Brichory F, Puravs E, Kuick R, Wood C, Rouillard JM, Tra J, Kardia S, Beer D, Hanash S*. 2001. *Univ Michigan, Med Ctr, 1500 West Med Ctr Dr, Med Sci Res Bldg 1, Ann Arbor, Mi 48109, USA. A database of protein expression in lung cancer. *Proteomics* **1:** (10) 1303.

Rickman DS, Bobek MP, Misek DE, Kuick R, Blaivas M, Kurnit DM, Taylor J, Hanash SM*. 2001. *Univ Michigan, Sch Med, Dept Pediat, Med Ctr, 1150 West Med Ctr Dr, Med Sci Res Bldg 1, Ann Arbor, Mi 48109, USA. Distinctive molecular profiles of high-grade and low-grade gliomas based on oligonucleotide microarray analysis. *Cancer Res* **61:** (18) 6885.

Rutherford RM, Kehren J, Staedtler F, Chibour SD, Egan JJG, Tamm M, Gilmartin JJ, Brutsche MH*. 2001. *Univ Basel Hosp, Dept Internal Med, Petersgraben 4, CH-4031 Basel, Switzerland. Functional genomics in sarcoidosis: Reduced or increased apoptosis? *Swiss Med Wkly* **131:** (31-32) 459.

Sreekumar A, Nyati MK, Varambally S, Barrette TR, Ghosh D, Lawrence TS, Chinnaiyan AM*. 2001. *Univ Michigan, Med Sch, Dept Pathol, 1301 Catherine Rd, Ann Arbor, Mi 48109, USA. Profiling of cancer cells using protein microarrays: Discovery of novel radiation-regulated proteins. *Cancer Res* **61:** (20) 7585.

Tripodis N, Hart AAM, Fijneman RJA, Demant P*. 2001. *Netherlands Canc Inst, Div Mol Genet-H5, Plesmanlaan 121, NL-1066 CX Amsterdam, The Netherlands. Complexity of lung cancer modifiers: Mapping

Copyright © 2002 John Wiley & Sons, Ltd. 

of thirty genes and twenty-five interactions in half of the mouse genome. *J Nat Cancer Inst* **93:** (19) 1484.

Watts GS, Futscher BW, Isett R, Gleason-Guzman M, Kunkel MW, Salmon SE. 2001. Univ Arizona, Arizona Canc Ctr, 1515 Nth Campbell Ave, Tucson, Az 85724, USA. cDNA microarray analysis of multidrug resistance: Doxorubicin selection produces multiple defects in apoptosis signaling pathways. *J Pharmacol Exp Ther* **299:** (2) 434.

Yowe D, Cook WJ, Gutierrez-Ramos JC. 2001. Millennium Pharmaceut, 45 Sydney St, Cambridge, Ma 02139, USA. Microarrays for studying the host transcriptional response to microbial infection and for the identification of host drug targets. *Microbes Infect* **3:** (10) 813.

### 8 EST, cDNA and other clone resources

Crookshanks M, Emmersen J, Welinder KG*, Nielsen KL. 2001. *Univ Aalborg, Inst Bioteknol, Sohngaardsholmsvej 49, DK-9000 Aalborg, Denmark. The potato tuber transcriptome: Analysis of 6077 expressed sequence tags. *FEBS Lett* **506:** (2) 123.

Holz C, Lueking A, Bovekamp L, Gutjahr C, Bolotina N, Lehrach H, Cahill DJ*. 2001. *Max-Planck-Inst Mol Genet, Ihnestr 73, DE-14195 Berlin, Germany. A human cDNA expression library in yeast enriched for open reading frames. *Genome Res* **11:** (10) 1730.

Sasaki Y, Asamizu E, Shibata D, Nakamura Y, Kaneko T, Awai K, Amagai M, Kuwata C, Tsugane T, Masuda T, Masuda T, Shimada H, Takamiya K, Ohta H*, Tabata S. 2001. *Tokyo Inst Technol, Grad Sch Biosci & Biotechnol, Dept Biol Sci, Midori ku, 4259 Nagatsuta, Yokohama, Kanagawa 226 850, Japan. Monitoring of methyl jasmonate-responsive genes in *Arabidopsis* by cDNA macroarray: Self-activation of jasmonic acid biosynthesis and crosstalk with other phytohormone signaling pathways. *DNA Res* **8:** (4) 153.

Snijders AM, Nowak N, Segraves R, Blackwood S, Brown N, Conroy J, Hamilton G, Hindle AK, Huey B, Kimura K, Law S, Myambo K, Palmer J, Ylstra B, Yue JP, Gray JW, Jain AN, Pinkel D, Albertson DG*. 2001. *Univ Calif, Ctr Comprehens Canc, San Francisco, Ca 94143, USA. Assembly of microarrays for genome-wide measurement of DNA copy number. *Nat Genet* **29:** (3) 263.

Wang YH, Garvin DF, Kochian LV*. 2001. *Cornell Univ, USDA/ARS, US Plant Soil & Nutr Lab, Ithaca, NY 14853, USA. Nitrate-induced genes in tomato roots. Array analysis reveals novel genes that may play a role in nitrogen nutrition. *Plant Physiol* **127:** (1) 345.

Woo YM, Hu DWN, Larkins BA*, Jung R. 2001. *Univ Arizona, Dept Plant Sci, Tucson, Az 85721, USA. Genomics analysis of genes expressed in maize endosperm identifies novel seed proteins and clarifies patterns of zein gene expression. *Plant Cell* **13:** (10) 2297.

### 9 Functional genomics

Birrell GW, Giaever G, Chu AM, Davis RW, Brown JM*. 2001. *Stanford Univ, Sch Med, Dept Radiat Oncol, Div Radiat & Canc Biol, 269 Campus Dr, Stanford, Ca 94305, USA. A genome-wide screen in *Saccharomyces cerevisiae* for genes affecting UV radiation sensitivity. *Proc Natl Acad Sci U S A* **98:** (22) 12608.

De Gregorio E, Spallman PT, Rubin GM, Lemaitre B*. 2001. *CNRS, Ctr Genet Mol, FR-91198 Gif-sur-Yvette, France. Genome-wide analysis of the *Drosophila* immune response by using oligonucleotide microarrays. *Proc Natl Acad Sci U S A* **98:** (22) 12590.

De Groot PWJ, Ruiz C, Vazquez-de Aldana CR, Duenas E, Cid VJ, Del Rey F, Rodriquez-Pena JM, Perez P, Andel A, Caubin J, Arroyo J, Garcia JC, Gil C, Molina M, Garcia LJ, Nombela C, Klis FM*. 2001. *Univ Amsterdam, Swammerdam Inst Life Sci, Nieuwe Achtergracht 166, NL-1018 WV Amsterdam, The Netherlands. A genomic approach for the identification and classification of genes involved in cell wall formation and its regulation in *Saccharomyces cerevisiae*. *Comp Funct Genom* **2:** (3) 124.

Dempsey AA, Dzau VJ, Liew CC*. 2001. *Harvard Univ, Brigham & Women's Hosp, Sch Med, Cardiovasc Genome Unit, 75 Francis St, Boston, Ma 02115, USA. Cardiovascular genomics: Estimating the total number of genes expressed in the human cardiovascular system. *J Mol Cell Cardiol* **33:** (10) 1879.

Grigoriev A. 2001. GPC Biotech, Fraunhoferstr 20, DE-82152 Martinsried, Germany. A relationship between gene expression and protein interactions on the proteome scale: Analysis of the bacteriophage T7 and the yeast *Saccharomyces cerevisiae*. *Nucleic Acids Res* **29:** (17) 3513.

Hegyi H, Gerstein M*. 2001. *Yale Univ, Dept Mol Biophys & Biochem, POB 6666, New Haven, Ct 06520, USA. Annotation transfer for genomics: Measuring functional divergence in multi-domain proteins. *Genome Res* **11:** (10) 1632.

Kohli A, Xiong J, Greco R, Christou P, Pereira A*. 2001. *Plant Res Int, Business Unit Genomics, POB 16, NL-6700 AA Wageningen, The Netherlands. Tagged Transcriptome Display (TTD) in *indica* rice using *Ac* transposition. *Mol Genet Genomics* **266:** (1) 1.

Rosenquist M, Alsterfjord M, Larsson C, Sommarin M. 2001. Lund Univ, Dept Plant Biochem, POB 117, SE-22100 Lund, Sweden. Data mining the *Arabidopsis* genome reveals fifteen 14-3-3 genes. Expression is demonstrated for two out of five novel genes. *Plant Physiol* **127:** (1) 142.

Willson TM, Jones SA, Moore JT, Kliewer SA. 2001. GlaxoSmithKline, Nucl Receptor Discovery Res, 5 Moore Dr, Res Triangle Park, NC 27709, USA. Chemical genomics: Functional analysis of orphan nuclear receptors in the regulation of bile acid metabolism. *Med Res Rev* **21:** (6) 513.

Wood V, Rutherford KM, Ivens A, Rajandream MA, Barrell B. 2001. Wellcome Trust Genome Campus, Sanger Ctr, Hinxton, Cambridge CB10 1SA, England. A re-annotation of the *Saccharomyces cerevisiae* genome. *Comp Funct Genom* **2:** (3) 143.

### 10 Transcriptomics

Boeuf S, Klingenspor M, Van Hal NLW, Schneider T, Keijer J, Klaus S*. 2001. *Deutsch Inst Ernahrungsforsch, Arthur Scheunert Allee 114-116, DE-14558 Bergholz Rehbrucke, Germany. Differential gene expression in white and brown preadipocytes. *Physiol Genomics* **7:** (1) 15.

Camargo AA, Samaia HP, Dias-Neto E, Simao DF, Migotto IA, Briones MR, Costa FF, Nagai MA, Verjovski-Almeida S, Zago MA *et al*. 2001. c/o Simpson AJG, Ludwig Inst Canc Res, BR-01509-010 Sao Paulo, Brazil. The contribution of 700,000 ORF sequence tags to the definition of the human transcriptome. *Proc Natl Acad Sci U S A* **98:** (21) 12103.

Cao D, Kocabas A, Ju Z, Karsi A, Li P, Patterson A, Liu Z*. 2001. *Auburn Univ, Dept Fisheries & Allied Aquacultures, Fish Mol Genet & Biotechnol Lab, Auburn, Al 36849, USA. Transcriptome of channel catfish (*Ictalurus punctatus*): Initial analysis of genes and expression profiles of the head kidney. *Anim Genet* **32:** (4) 169.

Chen BPC, Li YS, Zhao YH, Chen KD, Li S, Lao JM, Yuan SL, Shyy JYJ, Chien S*. 2001. *Univ Calif San Diego, Dept Bioengn, Mail Code 0427, 9500 Gilman Dr, La Jolla, Ca 92093, USA. DNA microarray analysis of gene expression in endothelial cells in response to 24-h shear stress. *Physiol Genomics* **7:** (1) 55.

Desikan R, Mackerness SAH, Hancock JT, Neill SJ*. 2001. *Univ West England, Ctr Res Plant Sci, Coldharbour Lane, Bristol BS16 1QY, England. Regulation of the *Arabidopsis* transcriptome by oxidative stress. *Plant Physiol* **127:** (1) 159.

Dinh HKB, Zhao BT, Schuschereba ST, Merrill G, Bowman PD*. 2001. *US Army, Inst Surg Res, 3400 Rawley E Chambers Ave, Bldg 3611, Ft Sam Houston, Tx 78234, USA. Gene expression profiling of the response to thermal injury in human cells. *Physiol Genomics* **7:** (1) 3.

Dupont J, Khan J, Qu BH, Metzler P, Helman L, Le Roith D*. 2001. *NIH/NIDDKD, Sect Cellular & Mol Physiol, Clin Endocrinol Branch, Room 8D12, Bldg 10, Bethesda, Md 20892, USA. Insulin and IGF-1 induce different patterns of gene expression in mouse fibroblast NIH-3T3 cells: Identification by cDNA microarray analysis. *Endocrinology* **142:** (11) 4969.

Ehrt S, Schnappinger D, Bekiranov S, Drenkow J, Shi SP, Gingeras TR, Gaasterland T, Schoolnik G, Nathan C*. 2001. *Cornell Univ, Weill Med Coll, Dept Microbiol & Immunol, 1300 York Ave, Box 57, New York, NY 10021, USA. Reprogramming of the macrophage transcriptome in response to interferon-$\gamma$ and *Mycobacterium tuberculosis*: Signaling roles of nitric oxide synthase-2 and phagocyte oxidase. *J Exp Med* **194:** (8) 1123.

Eszlinger M, Krohn K, Paschke R*. 2001. *Univ Leipzig, Dept Med III, PH Rosenthal Str 27, DE-04103 Leipzig, Germany. Complementary DNA expression array analysis suggests a lower expression of signal transduction proteins and receptors in cold and hot thyroid nodules. *J Clin Endocrinol Metab* **86:** (10) 4834.

Fletcher ST, Baker VA, Fentem JH, Basketter DA, Kelsell DP. 2001.

Copyright © 2002 John Wiley & Sons, Ltd.

Unilever Res, SEAC Toxicol Unit, Sharnbrook MK44 1LQ, England. Gene expression analysis of EpiDerm™ following exposure to SLS using cDNA microarrays. *Toxicol Vitro* **15:** (4-5) 393.

Harries HM, Fletcher ST, Duggan CM, Baker VA. 2001. Unilever Res, Colworth House, SEAC Toxicol Unit, Sharnbrook MK44 1LQ, England. The use of genomics technology to investigate gene expression changes in cultured human liver cells. *Toxicol Vitro* **15:** (4-5) 399.

Lee YT, Miller LD, Gubin AN, Makhlouf F, Wojda U, Barrett AJ, Liu ET, Miller JL*. 2001. *NIH/NIDDKD, Biol Chem Lab, Bldg 10, Rm 9817, Bethesda, Md 20892, USA. Transcription patterning of uncoupled proliferation and differentiation in myelodysplastic bone marrow with erythroid-focused arrays. *Blood* **98:** (6) 1914.

Mariani L, Beaudry C, McDonough WS, Hoelzinger DB, Demuth T, Ross TR, Bevens T, Coons SW, Watts G, Trent JM, Wei JS, Giese A, Berens ME . 2001. NRC Bldg, 4th Floor, 350 West Thomas Rd, Phoenix, Az 85013, USA. Glioma cell motility is associated with reduced transcription of proapoptotic and proliferation genes: A cDNA microarray analysis. *J Neurooncol* **53:** (2) 161.

Muhle RA, Pavlidis P, Grundy WN, Hirsch E*. 2001. *Northwestern Univ, Div Obstet, 2650 Ridge Ave, Evanston, Il 60201, USA. A high-throughput study of gene expression in preterm labor with a subtractive microarray approach. *Am J Obstet Gynecol* **185:** (3) 716.

Pillai B, Brahmachari SK, Sadhale PP*. 2001. *Indian Inst Sci, Dept Microbiol & Cell Biol, IN-560012 Bangalore, India. Genome-wide expression profile of RNA polymerase II subunit mutant of yeast using microarray technology. *Curr Sci* **81:** (5) 574.

Ragno S, Romano M, Howell S, Pappin DJC, Jenner PJ, Colston MJ*. 2001. *Natl Inst Med Res, Div Mycobacterial Res, Mill Hill, London NW7 1AA, England. Changes in gene expression in macrophages infected with *Mycobacterium tuberculosis*: A combined transcriptomic and proteomic approach. *Immunology* **104:** (1) 99.

Saban R, Saban MR, Nguyen NB, Hammond TG, Wershil BK. 2001. Univ Oklahoma, Hlth Sci Ctr, Coll Med, Dept Physiol, 940 SL Young Blvd, Rm 605, Oklahoma City, Ok 73104, USA. Mast cell regulation of inflammation and gene expression during antigen-induced bladder inflammation in mice. *Physiol Genomics* **7:** (1) 35.

Sampson NS, Ryan ST, Enke DA, Cosgrove D*, Koteliansky V, Gotwals P. 2001. *Biogen Inc, 14 Cambridge Ctr, Cambridge, Ma 02142, USA. Global gene expression analysis reveals a role for the $\alpha_1$ integrin in renal pathogenesis. *J Biol Chem* **276:** (36) 34182.

Van Dyk TK, DeRose EJ, Gonye GE. 2001. DuPont Co CR&D, Rt 141 & Powdermill Rd, POB 80173, Wilmington, De 19880, USA. LuxArray, a high-density, genomewide transcription analysis of *Escherichia coli* using bioluminescent reporter strains. *J Bacteriol* **183:** (19) 5496.

Vawter MP, Barrett T, Cheadle C, Sokolov BP, Wood WH, Donovan DM, Webster M, Freed WJ, Becker KG. 2001. Univ Calif Irvine, Coll Med, Dept Psychiat & Human Behav, Med Sci, Irvine, Ca 92697, USA. Application of cDNA microarrays to examine gene expression differences in schizophrenia. *Brain Res Bull* **55:** (5) 641.

Xiao JZ, Gregersen S, Kruhoffer M, Pedersen SB, Orntoft TF, Hermansen K*. 2001. *Aarhus Univ Hosp, Dept Endocrinol & Metab C, Tage Hansens Gade 2, DK-8000 Aarhus C, Denmark. The effect of chronic exposure to fatty acids on gene expression in clonal insulin-producing cells: Studies using high density oligonucleotide microarray. *Endocrinology* **142:** (11) 4777.

Yano N, Habib NA*, Fadden KJ, Yamashita H, Mitry R, Jauregui H, Kane A, Endoh M, Rifai A. 2001. *Univ London, Imperial Coll Sci & Technol, Sch Med, Div Surg Anaesthet & Intens Care, London W12 0NN, England. Profiling the adult human liver transcriptome: Analysis by cDNA array hybridization. *J Hepatol* **35:** (2) 178.

### 11 Proteomics

Arrell DK, Neverova I, Fraser H, Marban E, Van Eyk JE*. 2001. *Queen's Univ, Dept Physiol, Room 429, Botterell Hall, Kingston, Ontario, Canada K7L 3N6. Proteomic analysis of pharmacologically preconditioned cardiomyocytes reveals novel phosphorylation of myosin light chain 1. *Circ Res* **89:** (6) 480.

Aulak KS, Miyagi M, Yan L, West KA, Massillon D, Crabb JW, Stuehr DJ*. 2001. *Cleveland Clin Fdn, Dept Immunol, 9500 Euclid Ave, Cleveland, Oh 44195, USA. Proteomic method identifies proteins nitrated *in vivo* during inflammatory challenge. *Proc Natl Acad Sci U S A* **98:** (21) 12056.

Buttner K, Bernhardt J, Scharf C, Schmid R, Mader U, Eymann C, Antelmann H, Volker A, Volker U, Hecker M*. 2001. *Univ Greifswald, Inst Mikrobiol, Friedrich Ludwig Jahn Str 15, DE-17487 Greifswald, Germany. A comprehensive two-dimensional map of cytosolic proteins of *Bacillus subtilis*. *Electrophoresis* **22:** (14) 2908.

Cagney G, Uetz P, Fields S*. 2001. *Univ Washington, Dept Genet, Box 357360, Seattle, Wa 98195, USA. Two-hybrid analysis of the *Saccharomyces cerevisiae* 26S proteasomes. *Physiol Genomics* **7:** (1) 27.

Champion KM, Nishihara JC, Joly JC, Arnott D. 2001. Genentech Inc, Dept Analyt Chem, MS 62, 1 DNA Way, Sth San Francisco, Ca 94080, USA. Similarity of the *Escherichia coli* proteome upon completion of different biopharmaceutical fermentation processes. *Proteomics* **1:** (9) 1133.

Chaurand P, Da Gue BB, Pearsall RS, Threadgill DW, Caprioli RM*. 2001. *Vanderbilt Univ Sch Med, 812 MRBI, Nashville, Tn 37232, USA. Profiling proteins from azoxymethane-induced colon tumors at the molecular level by matrix-assisted laser desorption/ionization mass spectrometry. *Proteomics* **1:** (10) 1320.

Consoli L, Damerval C*. 2001. *INRA/INA-PG/UPS, Stn Genet Vegetale, UMR 320, Ferme Moulon, FR-91190 Gif-sur-Yvette, France. Quantification of individual zein isoforms resolved by two-dimensional electrophoresis: Genetic variability in 45 maize inbred lines. *Electrophoresis* **22:** (14) 2983.

Dreger M, Bengtsson L, Schoneberg T, Otto H, Hucho F*. 2001. *Free Univ Berlin, Inst Chem Biochem, Thielallee 63, DE-14195 Berlin, Germany. Nuclear envelope proteomics: Novel integral membrane proteins of the inner nuclear membrane. *Proc Natl Acad Sci U S A* **98:** (21) 11943.

Giard JC, Laplace JM, Rince A, Pichereau V, Benachour A, Leboeuf C, Flahaut S, Auffray Y, Hartke A. 2001. Univ Caen, Lab Microbiol Environm, FR-14032 Caen, France. The stress proteome of *Enterococcus faecalis*. *Electrophoresis* **22:** (14) 2947.

Guo XX, Ying WT, Wan JH, Hu ZY, Qian XH, Zhang HW, He FC*. 2001. *Inst Radiat Med, Dept Genomics & Proteomics, 27 Taiping Rd, CN-100850 Beijing, Peoples Rep China. Proteomic characterization of early-stage differentiation of mouse embryonic stem cells into neural cells induced by all-*trans* retinoic acid *in vitro*. *Electrophoresis* **22:** (14) 3067.

Hirschberg D, Cederlund E, Crosas B, Jonsson A, Tryggvason S, Farres J, Pares X, Bergman T, Jornvall H*. 2001. *Karolinska Inst, Dept Med Biochem & Biophys, SE-17177 Stockholm, Sweden. N-terminal acetylation in a third protein family of vertebrae alcohol dehydrogenase/retinal reductase found through a 'proteomics' approach in enzyme characterization. *Cell Mol Life Sci* **58:** (9) 1323.

Huang CM, Shui HA, Wu YT, Chu PW, Lin KG, Kab LS, Chen ST*. 2001. *Acad Sinica, Inst Biol Chem, 128 Sec 2 Yan Chiu Yuan Rd, Taipei 11529, Taiwan. Proteomic analysis of proteins in PC12 cells before and after treatment with nerve growth factor: Increased levels of a 43-kDa chromogranin B-derived fragment during neuronal differentiation. *Mol Brain Res* **92:** (1-2) 181.

Imin N, Kerim T, Weinman JJ*, Rolfe BG. 2001. *Australian Natl Univ, Res Sch Biol Sci, Genomic Interact Grp, POB 475, Canberra, ACT 2601, Australia. Characterization of rice anther proteins expressed at the young microspore stage. *Proteomics* **1:** (9) 1149.

Jefferies JR, Campbell AM, Van Rossum AJ, Barrett J, Brophy PM. 2001. Univ Wales, Inst Biol Sci, Aberystwyth SY23 3DA, Wales. Proteomic analysis of *Fasciola hepatica* excretory-secretory products. *Proteomics* **1:** (9) 1128.

Jenkins RE, Hawley SR, Promwikorn W, Brown J, Hamlett J, Pennington SR*. 2001. *Univ Liverpool, New Med Sch, Dept Human Anat & Cell Biol, Liverpool L69 3GE, England. Regulation of growth factor induced gene expression by calcium signalling: Integrated mRNA and protein expression analysis. *Proteomics* **1:** (9) 1092.

Joshi DD, Dang AJ, Yadav P, Qian J, Bandari PS, Chen KH, Donnelly R, Castro T, Gascon P, Haider A, Rameshwar P*. 2001. *Univ Med & Dent New Jersey, New Jersey Med Sch, MSB, Informat Syst & Technol Acad Comp Ctr, Newark, NJ 07103, USA. Negative feedback on the effects of stem cell factor on hematopoiesis is partly mediated through neutral endopeptidase activity on substance P: A combined functional and proteomic study. *Blood* **98:** (9) 2697.

Joubert R, Strub JM, Zugmeyer S, Kobi D, Carte N, Van Dorsselaer A, Boucherie H, Jaquet-Gutfreund L*. 2001. *TEPRAL Res Ctr, Brasseries Kronenbourg, 68 Route Oberhausbergen, FR-67200 Strasbourg, France. Identification by mass spectrometry of two-dimensional gel

Copyright © 2002 John Wiley & Sons, Ltd. 

electrophoresis-separated proteins extracted from lager brewing yeast. *Electrophoresis* **22:** (14) 2969.

Jungblut PR. 2001. Max-Planck-Inst Infect Biol, Support Unit Biochem, Schumann Str 21-22, DE-10117 Berlin, Germany. Proteome analysis of bacterial pathogens. *Microbes Infect* **3:** (10) 831.

Kirkpatrick C, Maurer LM, Oyelakin NE, Yoncheva YN, Maurer R, Slonczewski JL*. 2001. *Kenyon Coll, Dept Biol, Gambier, Oh 43022, USA. Acetate and formate stress: Opposite responses in the proteome of *Escherichia coli*. *J Bacteriol* **183:** (2) 6466.

Konishi H, Ishiguro K, Komatsu S*. 2001. *Natl Inst Agrobiol Sci, Dept Mol Genet, 2-1-2 Kannondai, Tsukuba, Ibaraki 305 8602, Japan. A proteomics approach towards understanding blast fungus infection of rice grown under different levels of nitrogen fertilization. *Proteomics* **1:** (9) 1162.

Kuon W, Holzhutter HG, Appel H, Grolms M, Kollnberger S, Traeder A, Henklein P, Weiss E, Thiel A, Lauster R, Bowness P, Radbruch A, Kloetzel PM, Sieper J*. 2001. *Free Univ Berlin, Klin Benjamin Franklin, Dept Med, Div Rheumatol, Hindenburgdamm 30, DE-12200 Berlin, Germany. Identification of HLA-B27-restricted peptides from the *Chlamydia trachomatis* proteome with possible relevance to HLA-B27-associated diseases. *J Immunol* **167:** (8) 4738.

Mattow J, Jungblut PR*, Schaible UE, Mollenkopf HJ, Lamer S, Zimny Arndt U, Hagens K, Muller EC, Kaufmann SHE. 2001. *Max-Planck-Inst Infect Biol, Cent Support Unit Biochem, Schumannstr 21-22, DE-10117 Berlin, Germany. Identification of proteins from *Mycobacterium tuberculosis* missing in attenuated *Mycobacterium bovis* BCG strains. *Electrophoresis* **22:** (14) 2936.

Mehta A, Rosato YB*. 2001. *UNICAMP, CBMEG, POB 6010, BR-13083-910 Campinas, SP, Brazil. Differentially expressed proteins in the interaction of *Xanthomonas axonopodis* pv. *citri* with leaf extract of the host plant. *Proteomics* **1:** (9) 1111.

Meng FY, Cargile BJ, Miller LM, Forbes AJ, Johnson JR, Kelleher NL*. 2001. *Univ Illinois, Dept Chem, 1209 West Calif St, Urbana, Il 61801, USA. Informatics and multiplexing of intact protein identification in bacteria and the archaea. *Nat Biotechnol* **19:** (10) 952.

Mills K, Mills PB, Clayton PT, Johnson AW, Whitehouse DB, Winchester BG*. 2001. *UCL, Biochem Endocrinol & Metab Unit, Inst Child Hlth, Great Ormond St Hosp, London WC1N 1EH, England. Identification of $\alpha_1$-antitrypsin variants in plasma with the use of proteomic technology. *Clin Chem* **47:** (11) 2012.

Ou KL, Seow TK, Liang RCMY, Lee BW, Goh DLM, Chua KY, Chung MCM. 2001. Natl Univ Singapore, Ctr Bioproc Technol, 10 Med Dr, SG-117597 Singapore, Rep Singapore. Identification of a serine protease inhibitor homologue in Bird's Nest by an integrated proteomics approach. *Electrophoresis* **22:** (16) 3589.

Patricelli MP, Giang DK, Stamp LM, Burbaum JJ. 2001. ActivX Biosci, 11025 Nth Torrey Pine Rd, Suite 120, La Jolla, Ca 92037, USA. Direct visualization of serine hydrolase activities in complex proteomes using fluorescent active site-directed probes. *Proteomics* **1:** (9) 1067.

Rabilloud T, Heller M, Rigobello MP, Bindoli A, Aebersold R, Lunardi J. 2001. CEA Grenoble, DBMS/BECP, Lab Bioenerget Cellulaire & Pathol, 17 rue Martyrs, FR-38054 Grenoble 9, France. The mitochondrial antioxidant defence system and its response to oxidative stress. *Proteomics* **1:** (9) 1105.

Randic M, Witzmann R, Vracko M, Basak SC*. 2001. *Drake Univ, Dept Math & Comp Sci, Des Mones, Ia 50311, USA. On characterization of proteomics maps and chemically induced changes in proteomes using matrix invariants: Application to peroxisome proliferators. *Med Chem Res* **10:** (7-8) 456.

Rogowska-Wrzesinska A, Mose-Larsen P, Blomberg A, Gorg A, Roepstorff P, Norbeck J, Fey SJ. 2001. Univ Sthn Denmark, Ctr Proteome Anal Life Sci, Int Space Park, Forskerparken 10B, DK-5230 Odense M, Denmark. Comparison of the proteomes of three yeast wild type strains: CEN.PK2, FY1679 and W303. *Comp Funct Genom* **2:** (4) 207.

Schafer H, Nau K, Sickmann A, Erdmann R, Meyer HE. 2001. Ruhr Univ Bochum, Inst Physiol Chem, Proteinstrukturlabor, DE-44780 Bochum, Germany. Identification of peroxisomal membrane proteins of *Saccharomyces cerevisiae* by mass spectrometry. *Electrophoresis* **22:** (14) 2955.

Shetty J, Diekman AB, Jayes FCL, Sherman NE, Naaby-Hansen S, Flickinger CJ, Herr JC*. 2001. *Univ Virginia, Dept Cell Biol, Charlottesville, Va 22908, USA. Differential extraction and enrichment of human sperm surface proteins in a proteome: Identification of immunocontraceptive candidates. *Electrophoresis* **22:** (14) 3053.

Sinha P, Poland J, Schnolzer M, Celis JE, Lage H. 2001. Univ Klin Charite, Inst Laboratoriums Med & Pathobiochem, Schumannstr 20-21, DE-10117 Berlin, Germany. Characterization of the differential protein expression associated with thermoresistance in human gastric carcinoma cell lines. *Electrophoresis* **22:** (14) 2990.

Srinivasan S, Traini M, Herbert B, Sexton D, Harry J, Alexander H, Williams KL, Alexander S*. 2001. *Univ Missouri, Div Biol Sci, 303 Tucker Hall, Columbia, Mo 65211, USA. Proteomic analysis of a developmentally regulated secretory vesicle. *Proteomics* **1:** (9) 1119.

Stevens FJ. 2001. Argonne Natl Lab, Biosci Div, 9700 Sth Cass Ave, Argonne, Il 60439, USA. Caveat receptor: Proteomes on display. *Comb Chem High Throughput Scr* **4:** (7) 599.

Stulik J, Hernychova L, Porkertova S, Knizek J, Macela A, Bures J, Jandik P, Langridge JI, Jungblut PR. 2001. Purkyne Milit Med Acad, Inst Radiobiol & Immunol, Trebesska 1575, CZ-50001 Hradec Kralove, Czech Republic. Proteome study of colorectal carcinogenesis. *Electrophoresis* **22:** (14) 3019.

Thery C, Boussac M, Veron P, Ricciardi-Castagnoli P, Raposo G, Garin J, Amigorena S. 2001. Inst Curie, Unite 520, INSERM, 12 rue Lhomond, FR-75005 Paris, France. Proteomic analysis of dendritic cell-derived exosomes: A secreted subcellular compartment distinct from apoptotic vesicles. *J Immunol* **166:** (12) 7309.

Waghray A, Feroze F, Schober MS, Yao FY, Wood C, Puravs E, Krause M, Hanash S, Chen YQ*. 2001. *Wayne State Univ, Dept Pathol, 540 East Canfield, Detroit, Mi 48201, USA. Identification of androgen-regulated genes in the prostate cancer cell line LNCaP by serial analysis of gene expression and proteomic analysis. *Proteomics* **1:** (10) 1327.

Wait R, Gianazza E*, Eberini I, Sironi L, Dunn MJ, Gemeiner M, Miller I. 2001. *Univ Milan, Fac Farm, Dipt Sci Farmacol, via Balzaretti 9, IT-20133 Milan, Italy. Proteins of rat serum urine and cerebrospinal fluid: VI. Further protein identification and interstrain comparison. *Electrophoresis* **22:** (14) 3043.

Wan JH, Wang JL, Cheng HP, Yu YT, Xing GC, Qiu ZY, Qian XH, He FC*. 2001. *Chinese Natl Human Genome Ctr, Inst Radiat Med, Dept Genomics & Proteom, CN-100850 Beijing, Peoples Rep China. Proteomic analysis of apoptosis initiation induced by all-*trans* retinoic acid in human acute promyelocytic leukemia cells. *Electrophoresis* **22:** (14) 3026.

Westbrook JA, Yan JX, Wait R, Welson SY, Dunn MJ. 2001. Harefield Hosp, Imperial Coll Sch Med, Natl Heart & Lung Inst, Heart Sci Ctr, Harefield UB9 6JH, England. Zooming-in on the proteome: Very narrow-range immobilised pH gradients reveal more protein species and isoforms. *Electrophoresis* **22:** (14) 2865.

Wick LM, Quadroni M, Egli T*. 2001. *Swiss Fed Inst Environm Sci & Technol, POB 611, Uberlandstr 133, CH-8600 Dubendorf, Switzerland. Short- and long-term changes in proteome composition and kinetic properties in a culture of *Escherichia coli* during transition from glucose-excess to glucose-limited growth conditions in continuous culture and *vice versa*. *Environ Microbiol* **3:** (9) 588.

Yu LR, Shao XX, Jiang WL, Xu D, Chang YC, Xu YH, Xia QC*. 2001. *Chinese Acad Sci, Inst Biol Sci, Inst Biochem & Cell Biol, 320 Yue Yang Rd, CN-200031 Shanghai, Peoples Rep China. Proteome alterations in human hepatoma cells transfected with antisense epidermal growth factor receptor sequence. *Electrophoresis* **22:** (14) 3001.

Zeindl-Eberhart E, Klugbauer S, Dimitrijevic N, Jungblut PR, Lamer S, Rabes HM*. 2001. *Univ Munich, Inst Pathol, Thalkirchner Str 36, DE-80337 Munich, Germany. Proteome analysis of rat hepatomas: Carcinogen-dependent tumor-associated protein variants. *Electrophoresis* **22:** (14) 3009.

Zuber P. 2001. Oregon Grad Inst Sci & Technol, Dept Biochem & Mol Biol, 20000 NW Walker Rd, Beaverton, Or 97006, USA. A peptide profile of the *Bacillus subtilis* genome. *Peptides* **22:** (10) 1555.

### 12 Protein structural genomics

Andrade MA, Perez-Iratxeta C, Ponting CP. 2001. Eur Mol Biol Lab, Meyerhofstr 1, DE-69012 Heidelberg, Germany. Protein repeats: Structures, functions, and evolution. *J Struct Biol* **134:** (2-3) 117.

Bonneau R, Tsai J, Ruczinski I, Baker D*. 2001. *Univ Washington, Dept Biochem, Box 357350, Seattle, Wa 98195, USA. Functional inferences from blind *ab initio* protein structures predictions. *J Struct Biol* **134:** (2-3) 186.

Cottage A, Edwards YJK, Elgar G*. 2001. *Wellcome Trust Genome Campus, UK Human Genome Mapping Project Resource Ctr, Hinxton,

Copyright © 2002 John Wiley & Sons, Ltd. 

Cambridge CB10 1SB, England. SAND, a new protein family: From nucleic acid to protein structure and function prediction. *Comp Funct Genom* **2:** (4) 226.

D'Alfonso G, Tramontano A, Lahm A. 2001. Ist Ric Biol Mol P Angeletti, via Pontina km 30600, IT-00040 Pomezia, Italy. Structural conservation in single-domain proteins: Implications for homology modeling. *J Struct Biol* **134:** (2-3) 246.

Di Gennaro JA, Siew N, Hoffman BT, Zhang L, Skolnick J, Neilson LI, Fetrow JS*. 2001. *GeneFormatics Inc, 5830 Oberlin Dr, Suite 200, San Diego, Ca 92121, USA. Enhanced functional annotation of protein sequences *via* the use of structural descriptors. *J Struct Biol* **134:** (2-3) 232.

Grishin NV. 2001. Univ Texas, SW Med Ctr, Howard Hughes Med Inst, 5323 Harry Hines Blvd, Dallas, Tx 75390, USA. Fold change in evolution of protein structures. *J Struct Biol* **134:** (2-3) 167.

Liu JF, Rost B*. 2001. *Columbia Univ, Dept Biochem & Mol Biophys, CUBIC, 650 West 168 St, New York, NY 10032, USA. Comparing function and structure between entire proteomes. *Protein Sci* **10:** (10) 1970.

Lupas AN, Ponting CP, Russell RB*. 2001. *Eur Mol Biol Lab, Meyerhofstr 1, DE-69012 Heidelberg, Germany. On the evolution of protein folds: Are similar motifs in different protein folds the result of convergence, insertion, or relics of an ancient peptide world? *J Struct Biol* **134:** (2-3) 191.

O'Donoghue P, Amaro RE, Luthey-Schulten Z. 2001. Univ Illinois, Sch Chem Sci, Urbana, Il 61801, USA. On the structure of hisH: Protein structure prediction in the context of structural and functional genomics. *J Struct Biol* **134:** (2-3) 257.

Pawlowski K, Rychlewski L, Zhang BH, Godzik A. 2001. AstraZeneca R&D Ltd, SE-22187 Lund, Sweden. Fold predictions for bacterial genomes. *J Struct Biol* **134:** (2-3) 219.

Zweckstetter M, Bax A. 2001. NIH/NIDDK, Chem Phys Lab, Bethesda, Md 20892, USA. Single-step determination of protein substructures using dipolar couplings: Aid to structural genomics. *J Am Chem Soc* **123:** (38) 9490.

### 14 Genomic approaches to development

Margulies EH, Kardia SLR, Innis JW*. 2001. *Univ Michigan, Sch Med, Dept Human Genet, Ann Arbor, Mi 48109, USA. A comparative molecular analysis of developing mouse forelimbs and hindlimbs using Serial Analysis of Gene Expression (SAGE). *Genome Res* **11:** (10) 1686.

Robles P, Micol JL*. 2001. *Univ Miguel Hernandez, Div Genet, Campus de Elche, ES-03202 Alicante, Spain. Genome-wide linkage analysis of *Arabidopsis* genes required for leaf development. *Mol Genet Genomics* **266:** (1) 12.

### 15 Technological advances

Badock V, Steinhusen U, Bommert K, Otto A*. 2001. *Max-Delbruck-Ctr Mol Med, Dept Prot Chem, Robert Rossle Str 10, DE-13092 Berlin, Germany. Prefractionation of protein samples for proteome analysis using reversed-phase high-performance liquid chromatography. *Electrophoresis* **22:** (14) 2856.

Belov ME, Anderson GA, Angell NH, Shen YF, Tolic N, Udseth HR, Smith RD*. 2001. *Pacific NW Natl Labs, Environm Mol Sci Lab, POB 999, Richland, Wa 99352, USA. Dynamic range expansion applied to mass spectrometry based on data-dependent selective ion ejection in capillary liquid chromatography Fourier transform ion cyclotron resonance for enhanced proteome characterization. *Anal Chem* **73:** (21) 5052.

Broude NE, Woodward K, Cavallo R, Cantor CR, Englert D. 2001. Boston Univ, Ctr Adv Biotechnol, 36 Cummington St, Boston, Ma 02215, USA. DNA microarrays with stem-loop DNA probes: Preparation and applications. *Nucleic Acids Res* **29:** (19) U22.

Cramer R, Corless S. 2001. Ludwig Inst Cancer Res, 91 Riding House St, London W1W 7BS, England. The nature of collision-induced dissociation processes of doubly protonated peptides: Comparative study for the future use of matrix-assisted laser desorption/ionization on a hybrid quadrupole time-of-flight mass spectrometer in proteomics. *Rapid Commun Mass Spectrom* **15:** (22) 2058.

Dolan PL, Wu Y, Ista LK, Metzenberg RL, Nelson MA, Lopez GP*. 2001. *Univ New Mexico, Dept Chem & Nucl Engn, Farris Engn Ctr, Albuquerque, NM 87131, USA. Robust and efficient synthetic method for forming DNA microarrays. *Nucleic Acids Res* **29:** (21) U37.

Ericsson D, Ekstrom S, Nilsson J, Bergquist J, Marko-Varga G, Laurell T*. 2001. *Lund Inst Technol, Dept Elect Measurements, POB 118, SE-22100 Lund, Sweden. Downsizing proteolytic digestion and analysis using dispenser-aided sample handling and nanovial matrix-assisted laser/desorption ionization-target arrays. *Proteomics* **1:** (9) 1072.

Fehring V, Wandschneider S, Lohr M*. 2001. *Univ Heidelberg, Fak Klin Med Mannheim, Med Klin II, Dept Med II, Sekt Mol Gastroenterol, DE-68135 Mannheim, Germany. Physical markers for landmarking fluorescently stained gel that facilitate automated spot-picking. *Electrophoresis* **22:** (14) 2903.

Han DK, Eng J, Zhou HL, Aebersold R*. 2001. *Inst Syst Biol, 4225 Roosevelt Way NE, Seattle, Wa 98105, USA. Quantitative profiling of differentiation-induced microsomal proteins using isotope-coded affinity tags and mass spectrometry. *Nat Biotechnol* **19:** (10) 946.

Haskins WE, Wang ZQ, Watson CJ, Rostand RR, Witowski SR, Powell DH, Kennedy RT*. 2001. *Univ Florida, Dept Chem, Gainesville, Fl 32611, USA. Capillary LC-$MS^2$ at the attomole level for monitoring and discovering endogenous peptide in microdialysis samples collected *in vivo*. *Anal Chem* **73:** (21) 5005.

Huang JX, Mehrens D, Wiese R, Lee S, Tam SW, Daniel S, Gilmore J, Shi M, Lashkari D*. 2001. *Genometrix Inc, 2700 Res Forest Dr, The Woodlands, Tx 77381, USA. High-throughput genomic and proteomic analysis using microarray technology. *Clin Chem* **47:** (10) 1912.

Lin SH, Tornatore P, King D, Orlando R, Weinberger SR*. 2001. *Ciphergen Biosyst Inc, 6611 Dumbarton Circle, Fremont, Ca 94555, USA. Limited acid hydrolysis as a means of fragmenting proteins isolated upon ProteinChip® array surfaces. *Proteomics* **1:** (9) 1172.

Lind K, Kresse M, Muller RH*. 2001. *Free Univ Berlin, Dept Pharmaceut Technol, Kelchstr 31, DE-12169 Berlin, Germany. Evaluation of desorption of proteins adsorbed to hydrophilic surfaces by two-dimensional electrophoresis. *Proteomics* **1:** (9) 1059.

Madoz-Gurpide J, Wang H, Misek DE, Brichory F, Hanash SM*. 2001. *Univ Michigan, Sch Med, Dept Pediat, 1150 West Med Ctr Dr, Box 0656, Ann Arbor, Mi 48109, USA. Protein based microarrays: A tool for probing the proteome of cancer cells and tissues. *Proteomics* **1:** (10) 1279.

Miliotis T, Marko-Varga G, Nilsson J, Laurell T*. 2001. *Lund Inst Technol, Dept Elect Measurements, Box 118, SE-22100 Lund, Sweden. Development of silicon microstructures and thin-film MALDI target plates for automated proteomics sample identifications. *J Neurosci Methods* **109:** (1) 41.

Murayama K, Fujimura T, Morita M, Shindo N. 2001. Juntendo Univ, Sch Med, Div Biochem Anal, Cent Lab Med Sci, Bunkyo ku, 2-1-1 Hongo, Tokyo 113 8421, Japan. One-step subcellular fractionation of rat liver tissue using a Nycodenz density gradient prepared by freezing-thawing and two-dimensional sodium dodecyl sulfate electrophoresis profiles of the main fraction of organelles. *Electrophoresis* **22:** (14) 2872.

Nordhoff E, Ehelhofer V, Giavalisco P, Eickhoff H, Horn M, Przewieslik T, Theiss D, Schneider U, Lehrach H, Gobom J. 2001. Max-Planck-Inst Mol Genet, Dept Lehrach, Ihnestr 73, DE-14195 Berlin, Germany. Large-gel two-dimensional electrophoresis-matrix assisted laser desorption/ionization-time of flight-mass spectrometry: An analytical challenge for studying complex protein mixtures. *Electrophoresis* **22:** (14) 2844.

Smolka MB, Zhou HL, Purkayastha S, Aebersold R. 2001. Inst Syst Biol, 4225 Roosevelt Way, Suite 200, Seattle, Wa 98105, USA. Optimization of the isotope-coded affinity tag-labeling procedure for quantitative proteome analysis. *Anal Biochem* **297:** (1) 25.

Tabuchi M, Baba Y. 2001. Univ Tokushima, Fac Pharmaceut Sci, Dept Med Chem, Shomachi 1-78, Tokushima 770 8505, Japan. A separation carrier in high-speed proteome analysis by capillary electrophoresis. *Electrophoresis* **22:** (16) 3449.

Tsugita A, Miyazaki K, Nabetani T, Nozawa T, Kamo M, Kawakami T. 2001. Proteomics Res Lab, Amakubo 1-16-1, Tsukuba, Ibaraki 305 0005, Japan. Application of chemical selective cleavage methods to analyze post-translational modification in proteins. *Proteomics* **1:** (9) 1082.

Von Eggeling F, Junker K, Fiedler W, Wollscheid V, Durst M, Claussen U, Ernst G. 2001. Univ Jena, Inst Human Genet & Anthropol, Kollegiengasse 10, DE-07740 Jena, Germany. Mass spectrometry meets chip technology: A new proteomic tool in cancer research? *Elec-*

Copyright © 2002 John Wiley & Sons, Ltd.

*trophoresis* **22:** (14) 2898.

Von Eggeling F, Gawriljuk A, Fiedler W, Ernst G, Claussen U, Klose J, Romer I. 2001. Univ Jena, Inst Humangenet & Anthropol, Kollegiengasse 10, DE-07740 Jena, Germany. Fluorescent dual colour 2D-protein gel electrophoresis for rapid detection of differences in protein pattern with standard image analysis software. *Int J Mol Med* **8:** (4) 373.

Wehr T. 2001. LC GC, 859 Williamette Street, St Eugene, Or 97401, USA. Separation technology in proteomics. *LC GC North Am* **19:** (7) 702.

Winssinger N, Harris JL, Backes BJ, Schultz PG*. 2001. *Scripps Clin & Res Inst, Dept Chem, 10550 Nth Torrey Pines Rd, La Jolla, Ca 92037, USA. From split-pool libraries to spatially addressable microarrays and its application to functional proteomic profiling. *Angew Chem Int Ed* **40:** (17) 3152.

Yang MCK, Ruan QG, Yang JJ, Eckenrode S, Wu S, McIndoe RA, She JX*. 2001. *Univ Florida, Coll Med, Dept Pathol Immunol & Lab Med, Ctr Mammalian Genet & Diabet, Gainesville, Fl 32610, USA. A statistical method for flagging weak spots improves normalization and ratio estimates in microarrays. *Physiol Genomics* **7:** (1) 45.

Yefimov S, Yergey AL, Chrambach A*. 2001. *NIH/NICHHD, Macromol Anal Sect, Bldg 10, Rm 9D50, Bethesda, Md 20892, USA. Sequential electroelution and mass spectrometric identification on intact sodium dodecyl sulfate-proteins labeled with 5(6)-carboxy-fluorescein-*N*-hydroxysuccinimide ester. *Electrophoresis* **22:** (14) 2881.

Yeung KKC, Kiceniuk AG, Li L*. 2001. *Univ Alberta, Fac Sci, Dept Chem, Chem Bldg, Edmonton, Alberta, Canada T6G 2G2. Capillary electrophoresis using a surfactant-treated capillary coupled with offline matrix-assisted laser desorption/ionization mass spectrometry for high efficiency and sensitivity detection of proteins. *J Chromatogr A* **931:** (1-2) 153.

Zhang RJ, Sioma CS, Wang SH, Regnier FE*. 2001. *Purdue Univ, Dept Chem, West Lafayette, In 47907, USA. Fractionation of isotopically labeled peptides in quantitative proteomics. *Anal Chem* **73:** (21) 5142.

### 16 Bioinformatics

Blaschke C, Valencia A*. 2001. *Nat Ctr Biotech, CNB-CSIC, Protein Design Grp, ES-28049 Cantoblanco, Madrid, Spain. Can bibliographic pointers for known biological data be found automatically? Protein interactions as a case study. *Comp Funct Genom* **2:** (4) 196.

Brazma A, Vilo J. 2001. Eur Bioinformat Institute, Outstn Hinxton, Eur Mol Biol Lab, Wellcome Trust Genome Campus, Cambridge CB10 1SD, England. Gene expression data analysis. *Microbes Infect* **3:** (10) 823.

Carter RJ, Dubchak I, Holbrook SR*. 2001. *Lawrence Berkeley Natl Lab, Phys Biosci Div, Computat & Theoret Biol Dept, 1 Cyclotron Rd, Berkeley, Ca 94720, USA. A computational approach to identify genes for functional RNAs in genomic sequences. *Nucleic Acids Res* **29:** (19) 3928.

Chetouani F, Glaser P, Kunst F. 2001. Inst Pasteur, Dept Biol Mol, Lab Genom Microorganismes Pathogenes, 25 rue Dr Roux, F-75724 Paris 15, France. FindTarget: Software for subtractive genome analysis. *Microbiology* **147:** (10) 2643.

Coghlan A, MacDonaill DA*, Buttimore NH. 2001. *Univ Dublin, Trinity Coll, Dept Chem, Dublin 2, Rep Ireland. Representation of amino acids as five-bit or three-bit patterns for filtering protein databases. *Bioinformatics* **17:** (8) 676.

Colinge J, Feger G*. 2001. *Serono Pharmaceut Res Inst, CH Aulx 14, CH-1228 Plan-les-ouates, Switzerland. Detecting the impact of sequencing errors on SAGE data. *Bioinformatics* **17:** (9) 840.

Cox DG, Canzian F. 2001. Int Agcy Res Canc, Genome Anal Grp, FR-69008 Lyon, France. Genotype transposer: Automated genotype manipulation for linkage desequilibrium analysis. *Bioinformatics* **17:** (8) 738.

De Bakker PIW, Bateman A, Burke DF, Miguel RN, Mizuguchi K, Shi J, Shirai H, Blundell TL. 2001. Univ Cambridge, Dept Biochem, 80 Tennis Court Rd, Cambridge CB2 1GA, England. HOMSTRAD: Adding sequence information to structure-based alignments of homologous protein families. *Bioinformatics* **17:** (8) 748.

Demirev PA, Lin JS, Pineda FJ, Fenselau C. 2001. Univ Maryland, Dept Chem, College Park, Md 20742, USA. Bioinformatics and mass spectrometry for microorganism identification: Proteome-wide post-translational modifications and database search algorithms for characterization of intact *H. pylori*. *Anal Chem* **73:** (19) 4566.

Douguet D, Labesse G. 2001. Univ Montpellier I, Ctr Biochim Struct, CNRS, UMR 5048, INSERM, U554, 15 Ave Charles Flahault, FR-34060 Montpellier, France. Easier threading through web-based comparisons and cross-validations. *Bioinformatics* **17:** (8) 752.

Goodstadt L, Ponting CP. 2001. Univ Oxford, Dept Human Anat & Genet, MRC Funct Genet Unit, Sth Parks Rd, Oxford OX1 3QX, England. CHROMA: Consensus-based colouring of multiple alignments for publication. *Bioinformatics* **17:** (9) 845.

Holmes I, Bruno WJ. 2001. Los Alamos Natl Lab, Grp T10, POB 1663, Los Alamos, NM 87545, USA. Evolutionary HMMs: A Bayesian approach to multiple alignment. *Bioinformatics* **17:** (9) 803.

Hua SJ, Sun ZR*. 2001. *Tsing Hua Univ, Dept Biol Sci & Biotechnol, Inst Bioinformat, CN-100084 Beijing, Peoples Rep China. Support vector machine approach for protein subcellular localization prediction. *Bioinformatics* **17:** (8) 721.

Karplus K, Hu BR. 2001. Univ Calif, Santa Cruz, Ca 95064, USA. Evaluation of protein multiple alignments by SAM-T99 using the BAliBASE multiple alignment test set. *Bioinformatics* **17:** (8) 713.

Kowalski J. 2001. Harvard Univ, Sch Publ Hlth, Dept Biostat, Boston, Ma 02115, USA. A non-parametric approach to translating gene region heterogeneity associated with phenotype into location heterogeneity. *Bioinformatics* **17:** (9) 775.

Makalowska I, Ryan JF, Baxevanis AD*. 2001. *NIH/NHGRI, Genome Technol Branch, Bldg 49, Room 4A-22, Bethesda, Md 20892, USA. Gene Machine: Gene prediction and sequence annotation. *Bioinformatics* **17:** (9) 843.

Marti-Renom MA, Ilyin VA, Sali A*. 2001. *Rockefeller Univ, Pels Family Ctr Biochem & Struct Biol, Labs Mol Biophys, 1230 York Ave, New York, NY 10021, USA. DBAli: A database of protein structure alignments. *Bioinformatics* **17:** (8) 746.

Ning ZM, Cox AJ, Mullikin JC*. 2001. *Sanger Ctr, Informat Div, Wellcome Trust, Genome Campus, Cambridge CB10 1SA, England. SSAHA: A fast search method for large DNA databases. *Genome Res* **11:** (10) 1725.

Ota S, Li WH*. 2001. *Univ Chicago, Dept Ecol & Evolut, 1101 East 59th St, Chicago, Il 60637, USA. NJML+: An extension of the NJML method to handle protein sequence data and computer software implementations. *Mol Biol Evol* **18:** (11) 1983.

Pei JM, Grishin NV*. 2001. *Univ Texas, SW Med Ctr, Howard Hughes Med Inst, 5323 Harry Hines Blvd, Dallas, Tx 75390, USA. AL2CO: Calculation of positional conservation in a protein sequence alignment. *Bioinformatics* **17:** (8) 700.

Sakharkar MK, Tan TW, De Souza SJ. 2001. Natl Univ Singapore, Bioinformatics Ctr, SG-119260 Singapore, Rep Singapore. Generation of a database containing discordant intron positions in eukaryotic genes (MIDB). *Bioinformatics* **17:** (8) 671.

Salama JJ, Feldman HJ, Hogue CWV*. 2001. *MDS Proteomics Inc, 480 Univ Ave, Toronto, Ontario, Canada M5S 1A8. VISTRAJ: exploring protein conformational space. *Bioinformatics* **17:** (9) 851.

Skrabanek L, Campagne F*. 2001. *CUNY Mt Sinai Sch Med, Inst Computat Biomed, Box 1218, 1 Gustave L Levy Pl, New York, NY 10029, USA. TissueInfo: High-throughput identification of tissue expression profiles and specificity. *Nucleic Acids Res* **29:** (21) U1.

Varotto C, Richly E, Salamini F, Leister D*. 2001. *Max-Planck-Inst Zuchtungsforsch, Abt Pflanzenzuchtung & Ertragsphysiol, Carl Von Linne Weg 10, DE-50829 Cologne, Germany. GST-PRIME: A genome-wide primer design software for the generation of gene sequence tags. *Nucleic Acids Res* **29:** (21) 4373.

Xing LQ, Brendel V. 2001. Iowa State Univ Sci & Technol, Dept Zool & Genet, Ames, Ia 50011, USA. Multi-query sequence BLAST output examination with MuSeqBox. *Bioinformatics* **17:** (8) 744.

Yeung KY, Ruzzo WL. 2001. Univ Washington, Box 352350, Seattle, Wa 98195, USA. Principal component analysis for clustering gene expression data. *Bioinformatics* **17:** (9) 763.

Zdobnov EM, Apweiler R. 2001. European Bioinformat Inst, EMBL Outstn, Wellcome Trust Genome Campus, Cambridge CB10 1SD, England. InterProScan: An integration platform for the signature-recognition methods in InterPro. *Bioinformatics* **17:** (9) 847.

Zmasek CM, Eddy SR. 2001. Washington Univ, Sch Med, Dept Genet, Howard Hughes Med Inst, St Louis, Mo 63110, USA. A simple algorithm to infer gene duplication and speciation events on a gene tree. *Bioinformatics* **17:** (9) 821.

Copyright © 2002 John Wiley & Sons, Ltd. 
